# Universal Tumor Screening in Colorectal Cancer: Role of MMR Immunohistochemistry for Lynch Syndrome and Early-Onset CRC

**DOI:** 10.3390/cancers18162549

**Published:** 2026-08-08

**Authors:** Silvia Negro, Sara Lessio, Daniele Passeri, Andrea Baldo, Marco Scarpa, Angelo Paolo Dei Tos, Ganmaria Pennelli, Francesca Schiavi, Claudia Pinato, Matteo Fassan, Quoc Riccardo Bao, Francesca Bergamo, Sara Lonardi, Gaya Spolverato, Emanuele Damiano Luca Urso

**Affiliations:** 1 Third Surgery Unit, Department of Surgical, Oncological, and Gastroenterological Sciences, University of Padua, Via Giustiniani 2, 35128 Padua, Italy; silvia.negro@unipd.it (S.N.); sara.lessio@studenti.unipd.it (S.L.); daniele.passeri@studenti.unipd.it (D.P.); marco.scarpa@aopd.veneto.it (M.S.); quocriccardo.bao@unipd.it (Q.R.B.); gaya.spolverato@unipd.it (G.S.); edl.urso@unipd.it (E.D.L.U.); 2 Surgical Pathology and Cytopathology Unit, Department of Integrated Diagnostics, Azienda Ospedale-Università Padova, 35124 Padua, Italy; angelopaolo.deitos@unipd.it (A.P.D.T.); gianmaria.pennelli@unipd.it (G.P.); matteo.fassan@unipd.it (M.F.); 3 Familial Cancer Clinic and Oncoendocrinology, Veneto Institute of Oncology-Scientific Institute for Research, Hospitalization and Health Care, IOV-IRCCS, 35128 Padua, Italy; francesca.schiavi@ioveneto.it (F.S.); claudia.pinato@iov.veneto.it (C.P.); 4 Medical Oncology Unit 1, Veneto Institute of Oncology-Scientific Institute for Research, Hospitalization and Health Care, IOV-IRCCS, 35128 Padua, Italy; francesca.bergamo@iov.veneto.it (F.B.); sara.lonardi@iov.veneto.it (S.L.)

**Keywords:** colorectal cancer, Lynch syndrome, mismatch repair deficiency, universal tumor screening, early-onset colorectal cancer

## Abstract

**Background**: Mismatch repair deficiency (MMRd) is a central molecular determinant of colorectal cancer (CRC) biology, prognosis, and treatment response, and Universal Tumour Screening (UTS) is advocated for Lynch syndrome (LS) detection; yet real-world performance across clinical subgroups remains limited. We evaluated MMRd distribution and UTS-based LS detection in a large consecutive surgical cohort. **Methods**: We retrospectively analyzed 1022 consecutive CRC patients undergoing surgical resection at the University Hospital of Padua (2015–2023). MMR status was assessed by immunohistochemistry; MMRd cases underwent reflex BRAF mutation testing and, when available, MLH1 promoter methylation analysis, followed by germline multigene panel testing for suspected LS. Clinicopathological features were compared by MMR status, age at onset, and tumour location. **Results**: MMR testing was performed in 875 patients (85.6%), rising from 67.0% (2015–2017) to 97.4% (2021–2023). MMRd was identified in 139 tumors (15.9%) and was independently associated with age ≥ 70 years, colonic location, and stage 0–II. Of 22 patients with confirmed LS, 13 (59.1%) were newly identified through UTS; family history showed no significant univariate association with LS status and was not independently associated with MMRd after multivariable adjustment. MMRd prevalence was numerically higher in early- than late-onset CRC (20.0% vs. 15.4%), approaching significance after multivariable adjustment (OR 1.90, 95% CI 0.99–3.64; *p* = 0.054); hereditary syndromes were also more frequent in early-onset disease. MMRd was markedly rarer in rectal than colonic cancer (4.1% vs. 22.5%; *p* < 0.0001), though MMRd rectal cancers arose in younger patients. **Conclusions**: UTS identified a substantial proportion of LS carriers missed by age- or family-history criteria. The relationship between age and MMRd prevalence proved more nuanced than a simple comparison would suggest, reinforcing the value of universal over selective testing across the age spectrum, while MMRd rectal cancer shows a distinct younger profile relevant to immunotherapy-based organ preservation.

## Simple Summary

Some colorectal cancers are caused by defects in the DNA-repair system known as mismatch repair deficiency. Detecting these tumors matters clinically: they behave differently, often respond well to immunotherapy, and can reveal Lynch syndrome, an inherited condition that raises cancer risk for patients and their relatives. We reviewed more than 1000 patients who underwent surgery for colorectal cancer at a single institution over nine years. Universal tumor testing identified many Lynch syndrome carriers with no prior diagnosis, illustrating the additional diagnostic value of systematic tumour testing beyond family history-based referral. At first glance, mismatch repair deficiency was similarly common in younger and older patients. However, once other tumour characteristics were taken into account, younger age showed a possible link to mismatch repair deficiency, suggesting that age alone is an unreliable basis for selecting patients for testing. Younger patients also carried a higher overall burden of hereditary cancer syndromes, underscoring the importance of genetic counseling in this group. Our findings support systematic mismatch repair testing for every colorectal cancer patient.

## 1. Introduction

The clinical landscape of colorectal cancer (CRC) has been transformed by the recognition of mismatch repair deficiency (MMRd) as a central molecular determinant of tumour biology, prognosis, and therapeutic response. Approximately 10–20% of CRCs display MMRd, resulting from impaired function of a system normally maintained by MLH1, PMS2, MSH2, and MSH6 [1,2]. MMRd status can be reliably identified by immunohistochemistry (IHC) or PCR-based microsatellite instability (MSI) testing, which demonstrates high concordance across platforms [3,4,5]. Beyond its established prognostic value and association with resistance to fluoropyrimidine monotherapy, MMRd/MSI-H status now guides therapeutic decisions across disease stages, particularly in metastatic and locally advanced rectal cancer [6,7,8,9]. Anti–PD-1 agents are approved as first-line therapy in metastatic MMRd CRC and have demonstrated unprecedented efficacy, including pathological complete response and organ preservation in locally advanced rectal cancer, making accurate MMR classification at diagnosis both a diagnostic and therapeutic imperative [10,11,12,13,14]. Among MMRd tumors, approximately 10–15% are attributable to Lynch syndrome (LS), the most common hereditary CRC syndrome, caused by germline pathogenic variants (GPVs) in MMR genes and carrying major implications for cascade testing, surveillance, and cancer prevention in affected families [15,16,17]. The remaining MMRd CRCs are largely sporadic, driven by somatic MLH1 promoter hypermethylation, typically with BRAF V600E co-mutation, and require systematic differentiation from LS through workup [18,19,20,21].

Traditional clinical selection tools, including Amsterdam II criteria and revised Bethesda guidelines, fail to identify between 28% and 68% of LS carriers, underscoring the inadequacy of symptom- or family history–driven referral strategies [22,23,24]. Universal Tumour Screening (UTS), the systematic application of MMR testing to all newly diagnosed CRCs irrespective of age or family history, was developed to address this gap and has been endorsed by international guidelines and, in Italy, formally recommended since 2015 [25,26,27]. Despite growing implementation, real-world data on UTS performance and diagnostic yield across diverse clinical subgroups remain limited.

Two populations deserve particular attention. First, early-onset CRC (eoCRC, diagnosed at age ≤ 50) is increasing with heterogeneous trends across countries and is widely assumed to carry a disproportionate burden of hereditary disease [28,29,30,31]. Whether MMRd prevalence in eoCRC genuinely exceeds that of late-onset disease, and whether this justifies differential screening strategies, has not been rigorously evaluated in large real-world cohorts. Second, MMRd rectal cancer represents a clinically and therapeutically distinct, yet undercharacterized, subgroup: while accounting for a minority of MMRd tumors, it occupies a uniquely relevant position in the current era of immunotherapy-based organ preservation, with emerging evidence of extraordinary response rates to neoadjuvant anti–PD-1 therapy in this setting [11,32,33]. Nevertheless, the clinicopathological profile of MMRd rectal cancer, including its hereditary associations and biological differences from MMRd colon cancer, remains poorly defined, and management recommendations continue to be largely extrapolated from colonic and metastatic disease [34,35]. Against this background, this study aimed to: (1) compare the clinicopathological features of MMRd and MMR-proficient CRC in a consecutive real-world surgical cohort; (2) evaluate the performance of UTS in identifying LS, with particular attention to the limitations of family history as a selection criterion; (3) assess whether MMRd and LS prevalence differ between early-onset and late-onset CRC; and (4) explore whether MMRd rectal cancer represents a distinct clinicopathological subgroup with specific hereditary and therapeutic priority. Collectively, these analyses provide a real-world framework for optimizing MMR-based screening and referral strategies across CRC subgroups.

## 2. Materials and Methods

### 2.1. Patient Selection and Data Collection

All consecutive patients who underwent surgical resection for CRC at the University Hospital of Padua between 1 January 2015 and 31 December 2023 were included in this retrospective study. Clinicopathological data were extracted from the institutional CRC database (via the REDCap platform), which is prospectively maintained and updated at each patient discharge [36,37]. All pathology reports were systematically reviewed. The dataset included the following variables: age, sex, body mass index (BMI), American Society of Anesthesiologists (ASA) score, family history of CRC in first- and second-degree relatives, primary tumour location, histologic grade, vascular and perineural invasion, TNM stage, immunohistochemistry (IHC) results for MMR proteins, BRAF mutation status (in cases with MLH1/PMS2 loss at IHC), MLH1 promoter methylation status when available, and germline testing results when performed. Pathological staging was uniformly (re)classified for the entire cohort according to the 8th edition of the Union for International Cancer Control (UICC) TNM system. Cases originally staged under the 7th edition (patients operated before its implementation in January 2018) were reclassified to ensure consistency across the study period. Stage 0 was defined as carcinoma in situ/intramucosal carcinoma without submucosal invasion. Tumour location was categorized as proximal colon, from the caecum to the splenic flexure; distal colon, from the splenic flexure to the rectosigmoid junction; and rectum, defined as tumors located within 15 cm from the anal verge. Tumour grade was considered not assessable when reliable histopathological grading could not be performed; these cases (n = 88) were excluded from analyses involving tumour grade. Family history of CRC was stratified into three mutually exclusive categories: no family history (no affected first-degree relative), FDR (one affected first-degree relative), and FDR-plus (two or more affected relatives, including at least one first-degree relative). Patients with only second-degree affected relatives and no affected first-degree relatives were included in the no family history category. Family history data were unavailable for 74 patients (8.5% of the MMR-tested cohort); these patients were excluded from all analyses involving family history variables.

### 2.2. Immunohistochemistry and Molecular Analyses

**Figure 1:**
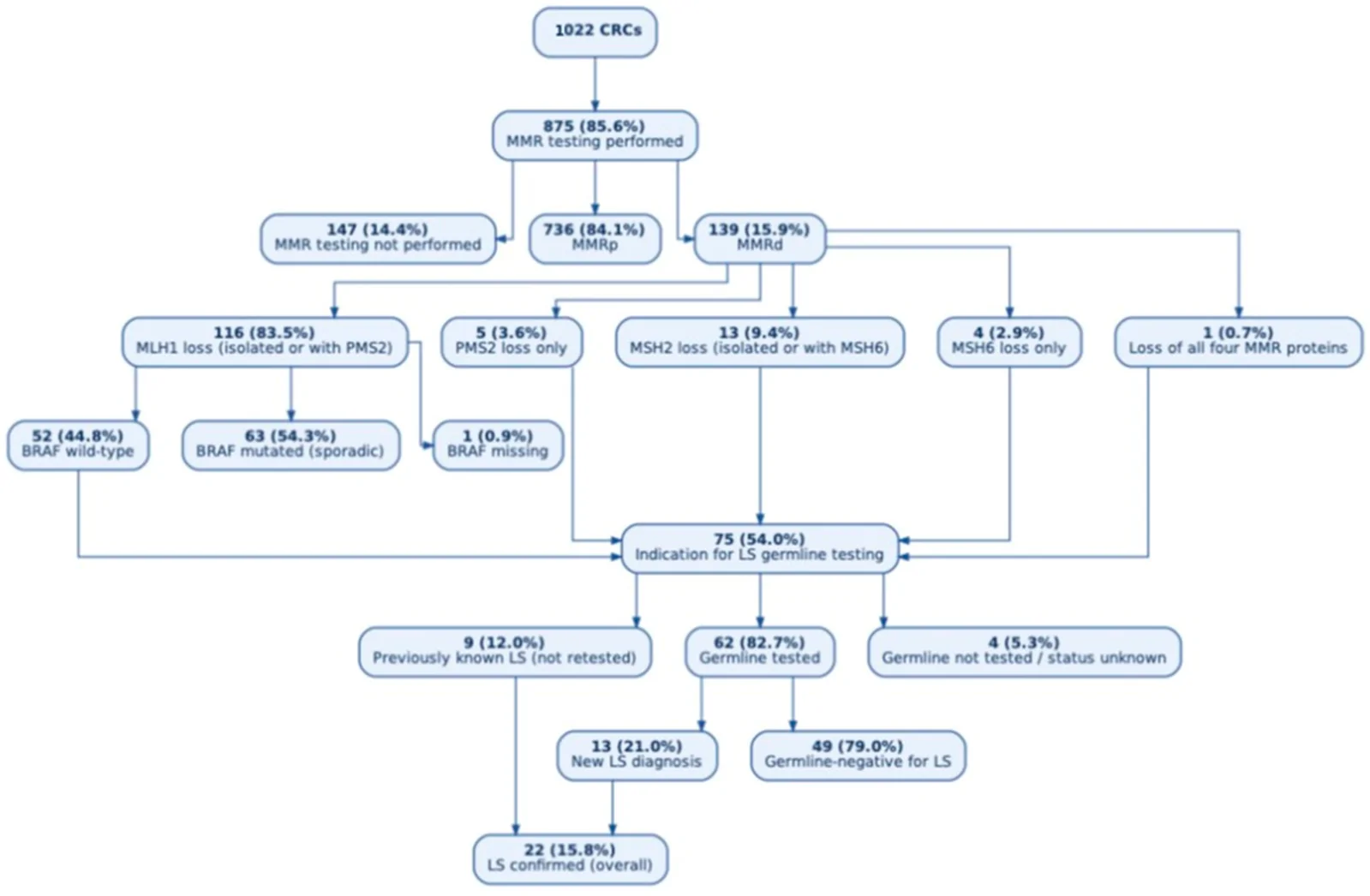
Diagnostic Workflow and Classification of MMRd Tumors in the Study Cohort.

MMR IHC was performed on formalin-fixed, paraffin-embedded (FFPE) tumour tissue sections using the Bond Polymer Refine Detection Kit (Leica Biosystems, Sheffield, UK) on the BOND-MAX automated platform. Sections of 4 μm were stained with monoclonal antibodies against MLH1 (clone ES05), PMS2 (clone EP51), MSH2 (clone FE11), and MSH6 (clone EP49) (all from Dako (Carpinteria, CA, USA)). Tumors were classified as MMR-deficient (MMRd) when complete loss of nuclear expression of at least one MMR protein was observed in tumour cells in the presence of retained internal positive control staining. All cases showing isolated MLH1 loss or combined MLH1/PMS2 loss underwent reflex BRAF V600E mutation analysis on FFPE tumour tissue. BRAF testing was performed using Sanger sequencing, Sequenom MassARRAY technology (Myriapod Colon Status, Diatech Pharmacogenetics (Jesi, Italy)), or the EasyPGX Real-Time BRAF kit (Diatech Pharmacogenetics), according to sample availability and assay implementation across the study period. In BRAF wild-type cases with MLH1 loss or combined MLH1/PMS2 loss, MLH1 promoter methylation analysis was subsequently performed by methylation-specific PCR, as part of the institutional universal tumour screening pathway implemented in accordance with Italian recommendations for hereditary CRC [26] (Figure S1). Cases with MLH1 loss and either BRAF V600E mutation or MLH1 promoter hypermethylation were classified as sporadic MMRd. Cases with MLH1 loss, wild-type BRAF, and absent MLH1 promoter hypermethylation were referred for genetic counselling and germline testing. Cases with isolated PMS2 loss, MSH2/MSH6 loss, isolated MSH2 loss, or isolated MSH6 loss were also referred for genetic counselling, irrespective of family history. Tumors with retained expression of all four MMR proteins were classified as MMRp. The complete universal tumour screening diagnostic pathway, including patient numbers at each step from IHC testing to germline confirmation, is shown in Figure 1. Patients with a previously known diagnosis of Lynch syndrome were included in the screening flowchart according to their tumour IHC profile but were not retested at the germline level. These patients were referred to the hereditary cancer multidisciplinary clinic for post-diagnostic management, surveillance planning, and cascade testing updates when appropriate.

### 2.3. Germline Testing

Patients were referred for genetic counselling and germline testing irrespective of personal or family cancer history in the presence of: (a) loss of MSH2, MSH6, or PMS2 expression on IHC; or (b) loss of MLH1 expression with wild-type BRAF and absence of MLH1 promoter hypermethylation. In addition, patients with early-onset CRC, defined as diagnosis at age ≤ 50 years, or with clinical or endoscopic suspicion of a polyposis syndrome were independently referred for genetic counselling irrespective of MMR status, following multidisciplinary evaluation. After written informed consent, peripheral blood was collected for genomic DNA extraction using the Promega Maxwell CSC system and the Maxwell^®^ CSC Whole Blood DNA Kit (Promega Corporation, Madison, WI, USA), according to the manufacturer’s instructions. Next-generation sequencing was performed using a custom multigene panel including the MMR genes MLH1, PMS2, MSH2, and MSH6, as well as EPCAM. Target enrichment of coding exons and flanking intronic regions of at least 25 bp across 81 hereditary cancer-associated genes was performed using hybridization capture with the Twist Library Prep EF Kit 2.0 and the custom capture panel TE-ERGI v2 (Twist Bioscience, San Francisco, CA, USA). Paired-end sequencing was performed on an Illumina MiSeq platform using v3 chemistry. Bioinformatic analysis was performed using Sophia DDM software v. 7.21.2 (SOPHiA GENETICS, Rolle (Switzerland HQ/Boston, MA (US HQ)) for the detection of single-nucleotide variants, insertions/deletions, and copy number variations. The NGS pipeline was validated in-house as a laboratory-developed test, with analytical sensitivity and specificity exceeding 99%. Variant nomenclature followed HGVS recommendations, and variant classification was based on ClinGen InSiGHT expert panel specifications aligned with ACMG/AMP criteria for MLH1, PMS2, MSH2, and MSH6. The pathogenic variant detection rate was defined as the proportion of patients with a confirmed pathogenic or likely pathogenic germline variant among those who underwent germline testing.

### 2.4. Statistical Analysis

Categorical data are presented as absolute numbers and percentages; continuous data as medians with interquartile ranges (IQRs). Categorical variables were compared using Fisher’s exact test or the χ^2^ test as appropriate; continuous variables were compared using the Wilcoxon rank-sum test. Univariate logistic regression models were used to examine associations between clinicopathological factors and MMRd status and LS-associated tumor status, respectively. Variables reaching statistical significance (*p* < 0.05) at univariate analysis were entered into a multivariable logistic regression model to identify independent predictors. To limit overfitting, the number of covariates included in each model was kept low relative to the number of outcome events; given the limited number of events for some outcomes (notably LS-associated tumors, n = 22), the corresponding multivariable models were considered exploratory and hypothesis-generating. To further assess whether early-onset status was independently associated with MMRd beyond the crude comparison, a dedicated multivariable logistic regression model was constructed with MMRd status as the outcome and early-onset status (≤50 years vs. >50 years) as the covariate of interest, adjusting for sex, tumour location, and stage. This model used the same ≤50-year threshold applied throughout the eoCRC/loCRC comparisons, independent of the ≥70-year cut-off used in the primary MMRd model. Given the descriptive and exploratory nature of these comparisons, *p*-values were not adjusted for multiple testing; associations of borderline significance should therefore be interpreted with caution. Model discrimination was assessed using the c-statistic (area under the receiver operating characteristic curve) with 95% confidence intervals; multicollinearity was evaluated using variance inflation factors (VIF), with values < 5 considered acceptable. Goodness-of-fit was additionally assessed using the Hosmer-Lemeshow test for the MMRd model; given the limited number of events, this test was not applied to the LS-associated status model, for which the events-per-variable ratio is reported instead. Logistic regression analyses were performed using R (version 4.3.1; R Foundation for Statistical Computing, Vienna, Austria); all other statistical analyses were conducted using GraphPad Prism (version 9.5.0). Missing data were handled by casewise deletion without imputation. Given that missing MMR data were predominantly concentrated in the early study period and likely reflect progressive implementation of UTS rather than random missingness, a pre-specified sensitivity analysis was performed restricting all primary analyses to patients operated between January 2018 and December 2023, a period characterized by near-complete MMR testing coverage (94.8%). All tests were two-sided with a significance level of *p* < 0.05.

### 2.5. Ethics

The study was approved by the Ethics Committee of the University Hospital of Padua (protocol number 007401, approved 24 November 2022) and conducted in accordance with the Declaration of Helsinki. Written informed consent for genetic counselling and germline testing was obtained from all patients who underwent germline analysis. For the retrospective collection and analysis of clinicopathological data, the Ethics Committee granted a waiver of specific informed consent, in view of the retrospective design and the inclusion of deceased or untraceable patients.

## 3. Results

### 3.1. Patient Characteristics

Between 2015 and 2023, 1022 consecutive CRC patients were included. Tumors were located in the proximal colon in 39.0%, the distal colon in 22.5%, and the rectum in 38.5% of cases. Overall, 117 patients (11.4%) were diagnosed at ≤50 years and classified as eoCRC. MMR testing was performed in 875 patients (85.6%), while it was unavailable in 147 cases (14.4%). When stratified by period of surgery, the proportion of patients without MMR testing progressively decreased over time, from 33.0% in 2015–2017 to 8.1% in 2018–2020 and 2.6% in 2021–2023, documenting the progressive real-world implementation of UTS in routine clinical practice. Baseline characteristics are reported in Table 1.

### 3.2. Clinicopathological Characteristics of MMRp and MMRd CRCs

Among the 875 patients with available MMR status, 139 tumors (15.9%) were classified as MMRd and 736 (84.1%) as MMRp. MMRd tumors occurred more frequently in females (58.9% vs. 42.6%, *p* = 0.0004) and in older patients (median age 76 vs. 70 years, *p* = 0.001). They were strongly enriched in the proximal colon (79.1% vs. 33.7%, *p* < 0.0001), more often diagnosed at early stages 0–II (64.0% vs. 51.1%, *p* < 0.0001), and more frequently classified as high-grade (51.8% vs. 22.6%, *p* < 0.0001). A family history of CRC in at least one first-degree relative was more common in MMRd cases (25.2% vs. 15.2%, *p* = 0.0035), and a strong family history (two or more affected relatives including at least one first-degree relative) was markedly enriched in MMRd tumors (7.9% vs. 1.8%, *p* < 0.0001). No significant differences were observed in BMI, vascular invasion, or perineural invasion. Complete clinicopathological data are shown in Table 2. At multivariable analysis, older age (≥70 years vs. <70 years; OR 2.41, 95% CI 1.04–5.63; *p* = 0.04), colonic location versus rectal location (OR 5.52, 95% CI 2.10–16.64; *p* = 0.001), and early tumour stage (stage 0–II vs. III–IV; OR 2.52, 95% CI 1.30–4.90; *p* = 0.006) remained independently associated with MMR deficiency. High-grade histology, strongly associated with MMRd at univariate level (OR 3.97, 95% CI 2.66–5.89; *p* < 0.0001), did not retain independent significance at multivariable analysis (OR 2.32, 95% CI 0.91–5.55; *p* = 0.06). Variance inflation factors excluded relevant statistical multicollinearity; this attenuation more likely reflects the close clinicopathological association between high-grade morphology, proximal tumour location, and MMRd status, whereby grading loses independent predictive value once location is accounted for in the model, rather than true statistical multicollinearity. Similarly, family history variables lost independent significance after adjustment. Full univariate and multivariable results are reported in Table 3. The multivariable model included 721 patients with complete data on all covariates, including 115 MMRd events (events-per-variable ratio 16.4). The multivariable MMRd model showed adequate discrimination (c-statistic 0.80, 95% CI 0.76–0.84) and acceptable calibration (Hosmer-Lemeshow *p* = 0.42), with variance inflation factors ranging from 1.05 to 1.47, confirming the absence of relevant multicollinearity among covariates.

### 3.3. Universal Tumour Screening and Lynch Syndrome Identification

**Figure 2:**
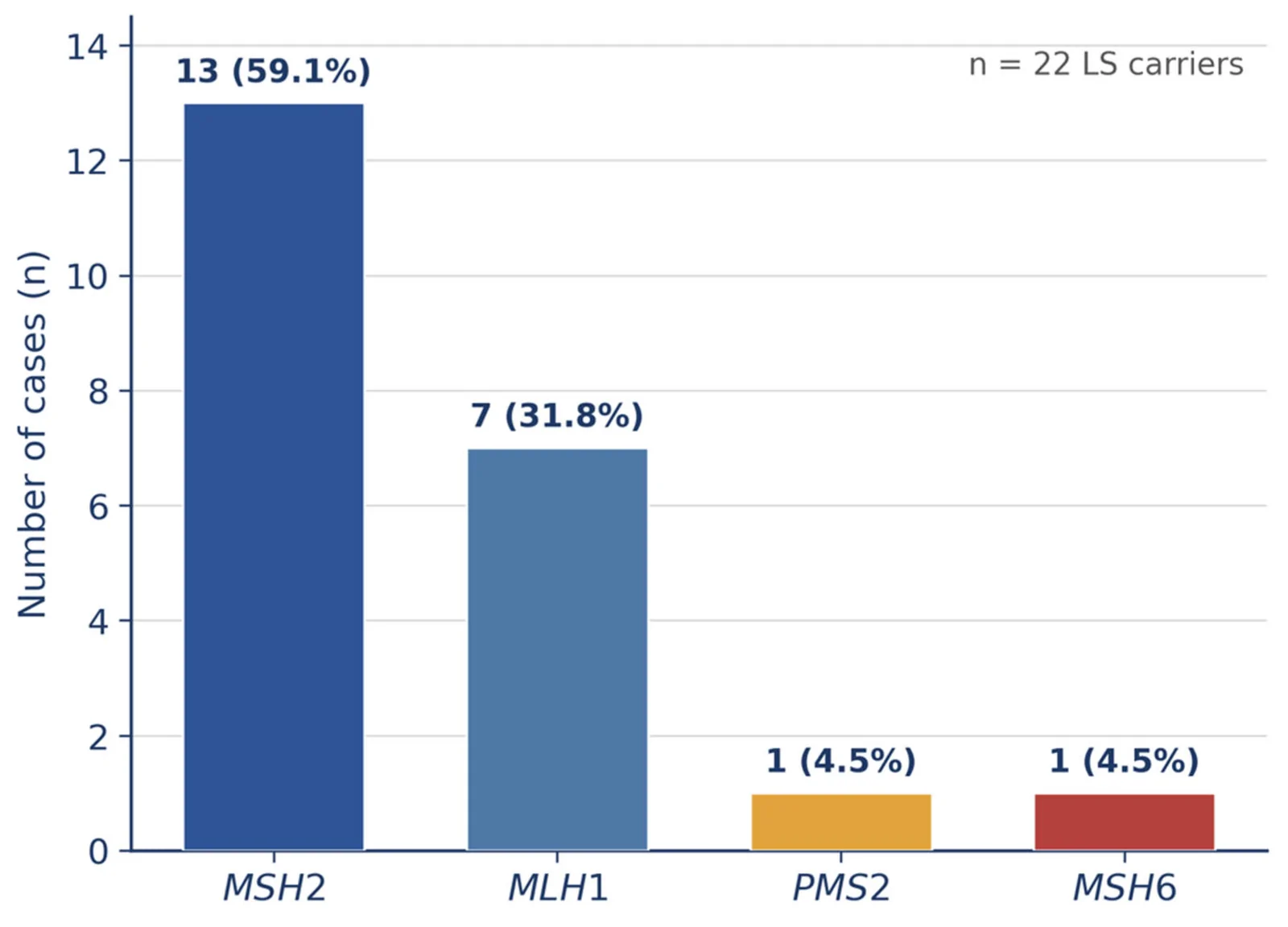
Distribution of germline pathogenic variants among patients with confirmed Lynch syndrome.

Among the 139 MMRd tumors, 116 cases (83.5%) showed loss of MLH1/PMS2. Among these MLH1/PMS2-deficient tumors, 52 were BRAF wild-type (44.8%), 63 were BRAF-mutated (54.3%), and 1 had missing BRAF status (0.9%). Less common IHC patterns included MSH2 loss, isolated or combined with MSH6 loss (13/139, 9.4%), isolated PMS2 loss (5/139, 3.6%), isolated MSH6 loss (4/139, 2.9%), and loss of all four MMR proteins (1/139, 0.7%). The complete UTS diagnostic pathway with patient numbers at each step is shown in Figure 1. Overall, 75 patients (54.0% of MMRd tumors) met the criteria for germline testing. Twenty-two patients with LS were resected for CRC: 9 (40.9%) had a previously known LS diagnosis, whereas 13 (59.1%) were newly identified through the UTS pathway. Excluding the previously known cases, 66 new patients were referred for germline testing on the basis of IHC and molecular workup. Germline testing was completed in 62 of 66 patients (93.9%), and a pathogenic variant was confirmed in 13 of 62 tested individuals (21.0% mutation detection rate). Across the whole LS cohort, the germline pathogenic variants most frequently involved MSH2 (13/22, 59.1%), followed by MLH1 (7/22, 31.8%), PMS2 (1/22, 4.5%), and MSH6 (1/22, 4.5%). A variant of uncertain significance (VUS) was identified in 7 of 62 tested individuals (11.3%); these patients were not counted as confirmed LS for the purposes of this analysis and were referred for standard clinical follow-up. The distribution of germline pathogenic variants is shown in Figure 2.

### 3.4. Clinicopathological Characteristics of Sporadic and LS-Associated MMRd CRCs

Among 139 MMRd tumors, 117 (84.2%) were classified as sporadic and 22 (15.8%) as LS-associated. Patients with LS-associated MMRd CRC were significantly younger than those with sporadic MMRd CRC (median age 50.5 vs. 77 years; *p* < 0.0001) and showed a less pronounced proximal colon predominance (54.5% vs. 83.8%; *p* = 0.001). A family history of CRC in first-degree relatives was numerically more frequent among LS-associated cases (36.4% vs. 23.1%), although this difference did not reach statistical significance (*p* = 0.17). No significant differences were observed in tumour stage, grading, vascular invasion, or perineural invasion between sporadic and LS-associated MMRd CRCs (Table 4). At multivariable analysis, age < 70 years was the only independent predictor of LS-associated MMRd CRC (OR 7.01, 95% CI 2.02–29.53; *p* = 0.003). Distal colon or rectal location, which was associated with LS status at univariate analysis (OR 4.29, 95% CI 1.66–11.71; *p* = 0.004), did not retain independent significance after adjustment (OR 1.54, 95% CI 0.34–6.17; *p* = 0.58) (Table 5). Given the limited number of events (n = 22), model discrimination rather than calibration was prioritized: the multivariable model included all 139 patients with MMRd CRC, and the LS-associated status model showed a c-statistic of 0.84 (95% CI 0.75–0.92), with an events-per-variable ratio of 11 (22 events/2 covariates). Variance inflation factors were approximately 1.1 for both covariates, indicating no relevant multicollinearity. The Hosmer-Lemeshow test was not applied given the limited event count.

### 3.5. Clinicopathological Characteristics of Early-Onset Colorectal Cancer (eoCRC) and Late-Onset CRC (loCRC)

Among patients with available MMR status, MMRd was detected in 19/95 eoCRC patients (20.0%) and 120/780 loCRC patients (15.4%; *p* = 0.23). To further evaluate this association independent of potential confounders, early-onset status was entered into a multivariable logistic regression model adjusting for sex, tumour location, and stage. After adjustment, the association between early-onset status and MMRd approached but did not reach conventional statistical significance (adjusted OR 1.90, 95% CI 0.99–3.64; *p* = 0.054), and was numerically stronger than the crude estimate (OR 1.37), consistent with partial confounding by the differential anatomical distribution of eoCRC tumours described below. Compared with loCRC, eoCRC tumors were more frequently located in the rectum (46.2% vs. 37.5%) and distal colon (28.2% vs. 21.8%), with a correspondingly lower proportion of proximal colon tumors (25.6% vs. 40.8%; *p* = 0.007). No significant differences were observed in tumour stage, grading, vascular invasion, perineural invasion, or first-degree CRC family history between eoCRC and loCRC. eoCRC patients had significantly lower ASA scores than loCRC patients (*p* < 0.0001), consistent with their younger age and lower comorbidity burden. Detailed comparisons are provided in Table 6. Hereditary CRC syndromes were identified in 15 eoCRC patients (12.8%) compared with 16 loCRC patients (1.8%), representing a significantly higher burden of germline predisposition in younger patients. Among all 31 carriers, Lynch syndrome was the most frequent diagnosis (n = 22; 11 eoCRC, 11 loCRC), followed by MUTYH-associated polyposis (n = 6; 3 eoCRC, 3 loCRC) and APC-associated polyposis (n = 3; 1 eoCRC, 2 loCRC). The higher absolute prevalence of hereditary syndromes in eoCRC is consistent with the known association between early-onset disease and germline predisposition and is likely further enriched in our cohort by the referral pattern of a tertiary center with an active hereditary CRC surveillance program.

### 3.6. Clinicopathological Characteristics of MMRd Colon and Rectal Cancers

MMRd status was markedly more prevalent in colon cancer than in rectal cancer (22.5% vs. 4.1%, *p* < 0.0001). Among MMRd tumors, patients with rectal primaries were significantly younger than those with colonic primaries (median age 67 vs. 77 years; *p* = 0.003) and were more frequently in the ≤50-year age group (38.5% vs. 11.1%; *p* = 0.03). Median BMI did not significantly differ between MMRd rectal and colon cancer patients (26.4 vs. 24.2 kg/m^2^; *p* = 0.23), whereas obesity was more frequent among MMRd rectal cancer patients (BMI ≥ 30: 46.2% vs. 14.3%; *p* = 0.01). Notably, no significant differences were observed in tumour stage, grading, vascular invasion, perineural invasion, or family history between MMRd colon and rectal cancers (Table 7).

### 3.7. Sensitivity Analysis

Results of the primary analyses were substantially consistent when restricted to the 686 patients operated between 2018 and 2023, a period characterized by near-complete UTS coverage (94.8%). MMRd prevalence in this subgroup was 16.6%, and the key associations between MMRd status and tumour location, age, and stage remained directionally consistent with the overall cohort findings, supporting the robustness of our results despite the higher proportion of missing MMR data in the early study period. Detailed sensitivity analysis results are provided in the Supplementary Materials Tables S1–S5.

## 4. Discussion

While the benefits of UTS for LS detection are established, real-world data quantifying its diagnostic yield beyond family history- or age-based selection remain scarce, particularly outside trial settings. Unlike prior series, which primarily confirm the feasibility of UTS or its yield in unselected cohorts, this study directly compares MMRd prevalence between early- and late-onset disease in a cohort of more than 1000 consecutive patients and separately characterizes MMRd rectal cancer as a distinct clinicopathological entity—a subgroup of growing relevance to immunotherapy-based organ preservation but previously undercharacterized outside metastatic or colonic MMRd populations. Our findings also reinforce why UTS outperforms selective strategies in practice, not only in principle. Of the 13 newly identified LS carriers, 8 (61.5%) were aged >50 years and had no first-degree family history of CRC; these patients, corresponding to 36.4% of all confirmed LS carriers in the cohort, would not have been selected using these simplified age- and CRC family history-based criteria. Three findings are of particular translational relevance: the complex relationship between MMRd prevalence and age at diagnosis, the substantial proportion of Lynch syndrome carriers identified through UTS independently of family history, and the distinct clinicopathological profile of MMRd rectal cancer in the context of emerging immunotherapy-based organ preservation strategies. A central finding of this study concerns the relationship between age at diagnosis and MMRd prevalence, which proved more nuanced than a simple comparison of raw proportions would suggest. The crude prevalence of MMRd was numerically higher in eoCRC than in loCRC (20.0% vs. 15.4%), but this comparison did not reach statistical significance and was underpowered (post hoc power ~20%; 95% CI for the absolute difference, −3.8% to +13.0%). Notably, after adjusting for tumour location, sex, and stage, the association between early-onset status and MMRd strengthened and approached conventional significance (adjusted OR 1.90, 95% CI 0.99–3.64; *p* = 0.054), numerically exceeding the crude estimate (OR 1.37). This pattern is consistent with partial confounding by the differential anatomical distribution of eoCRC tumours, which are disproportionately located in the rectum and distal colon (Table 6)—sites where MMRd is markedly less common than in the proximal colon (Table 2)—potentially masking a true underlying association in the unadjusted comparison. Taken together, these findings temper rather than support the assumption that younger age at CRC diagnosis is unrelated to MMR deficiency; instead, they suggest that the true relationship between age and MMRd may be obscured by anatomical distribution unless appropriately adjusted for, and that the crude comparison alone is insufficient to settle the question. Given the wide confidence interval and the modest number of events in the eoCRC subgroup, this adjusted association should be regarded as hypothesis-generating and warrants confirmation in larger cohorts. This finding complements observations at the opposite end of the age spectrum: Li et al. similarly demonstrated that raising the upper age limit for reflex MMR screening to 75–80 years would miss only 1.6–4.8% of LS cases, indicating that age-based thresholds—whether applied to younger or older patients—remain an imprecise substitute for direct MMR testing across the age spectrum [38]. Irrespective of the direction of the age association, the biological heterogeneity of eoCRC—encompassing hereditary syndromes, lifestyle-associated sporadic cancers, and a growing proportion of young-onset tumours with no identifiable hereditary etiology—is not fully captured by MMRd prevalence alone, and universal, unselected testing remains the only strategy that reliably identifies MMRd/LS regardless of age or anatomical site.

The overall MMRd prevalence of 15.9% in our cohort is consistent with large population-based and surgical series, confirming the expected distribution in an unselected CRC population [39,40]. MMRd tumors displayed the well-established clinicopathological signature of microsatellite-unstable cancers, proximal predominance, early-stage diagnosis, high-grade histology, and female preponderance, driven by hypermutability, frameshift neoantigen generation, and dense immune infiltration [19,39]. The marked enrichment of MMRd in the proximal colon (79.1% vs. 33.7% in MMRp, *p* < 0.0001) reflects both the embryological origin of the right colon and the differential epigenetic landscape of proximal colonic mucosa, which predisposes to MLH1 promoter hypermethylation-driven sporadic MMRd []. At multivariable analysis, colonic location, early tumour stage, and age ≥ 70 years independently predicted MMRd status. High-grade histology, strongly associated with MMRd at univariate level, lost independent significance despite the absence of relevant statistical multicollinearity (VIF 1.05–1.47), likely reflecting the close clinicopathological overlap between high-grade morphology, proximal location, and MMRd-associated mucinous and poorly differentiated tumour features. Importantly, while a strong family history of CRC was associated with MMRd on univariate analysis, it did not retain independent significance at multivariable modelling. This observation has direct clinical implications: it confirms that family history, even when present, is an insufficient surrogate for MMR status and cannot substitute for systematic tumour testing [24,41]. The converse is equally true: the absence of family history of CRC does not reliably exclude MMRd, further reinforcing the rationale for universal rather than selective screening. It should be emphasized that only family history for CRC is recorded in our database. If family history of other neoplasms included in the Amsterdam II criteria were also recorded, the performance of family history in including or excluding the possibility of LS, would likely be greater.

UTS implementation increased progressively over the study period, from 67.0% of cases in 2015–2017 to 97.4% in 2021–2023, documenting the real-world trajectory of guideline uptake at a tertiary referral center and providing a natural longitudinal framework for evaluating program maturation. The clinical impact of this implementation is substantial: among the 22 LS carriers enrolled in the study, 13 (59.1%) were newly diagnosed through the UTS pathway—individuals who carried no prior LS diagnosis and would have been missed under CRC family history– or age-based referral strategies alone. This proportion is consistent with, and at the higher end of, estimates from landmark UTS validation studies demonstrating that traditional clinical criteria fail to identify between 28% and 68% of LS carriers. Our data thus provide real-world confirmation, in a contemporary Italian surgical cohort, that UTS delivers a LS detection yield that is structurally inaccessible to selective testing approaches [27,42].

The germline pathogenic variant spectrum (MSH2 (59.1%), MLH1 (31.8%), PMS2 (4.5%), MSH6 (4.5%)) diverges from population-level LS registries, where MLH1 variants typically account for the plurality of cases [43,44]. The overrepresentation of MSH2 in our series most likely reflects tertiary center referral bias and the clinical enrichment of index cases with a suspected hereditary phenotype and should not be interpreted as reflecting the population-level distribution of LS variants. The overall germline confirmation rate of 21.0% among the newly tested patients falls within the range reported in comparable UTS cohorts and reflects the inherent composition of the group, which encompasses both true LS and sporadic MMRd cases with indeterminate methylation or BRAF results. This underscores the importance of complete sequential molecular workup, IHC, BRAF mutation analysis, and MLH1 promoter methylation testing before germline referral, both to maximize diagnostic yield and to avoid unnecessary genetic testing burden on patients and healthcare systems [21,45].

Among confirmed LS cases, patients were significantly younger than those with sporadic MMRd (median age 50.5 vs. 77 years, *p* < 0.0001) and showed a less pronounced proximal colon predominance (54.5% vs. 83.8%, *p* = 0.001), consistent with the known anatomical heterogeneity of LS-associated CRC compared to the predominantly right-sided distribution of sporadic MMRd. Family history of CRC was numerically more frequent in LS cases (36.4% vs. 23.1%), but this difference did not reach statistical significance (*p* = 0.17), a finding that, rather than reflecting a true biological equivalence, likely reflects the limited statistical power of this subgroup comparison and the well-documented incompleteness of family history data in clinical practice. At multivariable analysis, younger age (<70 years) was the only independent predictor of LS-associated MMRd (OR 7.01, 95% CI 2.02–29.53), while distal or rectal location lost independent significance after adjustment, indicating that the apparent topographic association between LS and distal CRC is largely confounded by age. Notably, the primary MMRd model identified older age (≥70 years) as an independent predictor, whereas the dedicated eoCRC model identified younger age (≤50 years) as showing a borderline association with MMRd; the two models address different age contrasts and, taken together, suggest a potentially non-linear relationship between age and MMRd that a simple dichotomization cannot fully capture. Taken together, these data reinforce the primacy of UTS applied universally, irrespective of age, anatomical location, or family history as the only reliable strategy for LS identification in routine clinical practice. Importantly, however, the overall burden of hereditary CRC syndromes was substantially higher in eoCRC than in loCRC (12.8% vs. 1.8%), with Lynch syndrome identified in 11 eoCRC patients, MUTYH-associated polyposis in 3, and APC-associated polyposis in 1. This broader germline predisposition in younger patients, extending well beyond LS and MMRd status, underscores the value of comprehensive multigene panel testing in eoCRC, independent of MMR results, and aligns with current international recommendations for expanded germline evaluation in early-onset disease [46,47]. The prevalence of hereditary syndromes in our eoCRC cohort (12.8%) is fully consistent with published estimates, which implicate hereditary syndromes in approximately 13% of eoCRC cases [48,49]. In the broader literature, approximately 28% of eoCRC patients report a family history of CRC [50,51,52], a proportion not significantly different from that observed in late-onset disease, underscoring that family history alone, whether from our data (18.8%) or external cohorts, remains an imprecise stratification tool. The distinct anatomical distribution of eoCRC, with enrichment in the rectum and distal colon compared to loCRC (*p* = 0.007), may further reflect underlying etiological differences and carries implications for endoscopic surveillance interval and modality in younger patients.

MMRd rectal cancer accounted for only 4.1% of rectal tumors in our cohort, compared with 22.5% of colon cancers (*p* < 0.0001), confirming the well-established anatomical gradient of MMRd across the colorectum and highlighting the rarity of this subgroup in routine surgical practice [33]. Despite its low prevalence, MMRd rectal cancer displayed a clinically distinct profile: patients were significantly younger than those with MMRd colon cancer (median age 67 vs. 77 years, *p* = 0.003) and more frequently classified as eoCRC (38.5% vs. 11.1%, *p* = 0.03) [36]. The younger age and higher eoCRC representation among MMRd rectal cancer patients suggest a proportionally greater contribution of LS-associated disease in this subgroup, consistent with the known predilection of LS for rectal and distal colorectal involvement and distinguishes MMRd rectal cancer biologically and clinically from its colonic counterpart [34,35]. The younger age observed among MMRd rectal cancers is biologically plausible rather than a likely artifact of sampling variability: LS-associated tumours, which are enriched among MMRd rectal cancers in our cohort, characteristically present at younger age than sporadic MLH1-methylated MMRd disease, which predominates in the proximal colon of older patients. This age skew is therefore consistent with the known hereditary enrichment of MMRd rectal cancer rather than a sampling artifact, although confirmation in larger rectal-specific cohorts is warranted given the modest subgroup size (n = 13). Notably, no significant differences in tumour stage, grading, vascular invasion, or perineural invasion were observed between MMRd colon and rectal cancers, indicating that the pathological aggressiveness of MMRd tumors does not differ meaningfully by anatomical site despite the differences in patient profile. A further distinguishing feature of MMRd rectal cancer was a higher prevalence of obesity: although median BMI did not differ significantly between MMRd rectal and colon cancers (26.4 vs. 24.2 kg/m^2^, *p* = 0.23), the proportion of obese patients (BMI ≥ 30 kg/m^2^) was markedly higher in the rectal subgroup (46.2% vs. 14.3%, *p* = 0.01). Given the small number of MMRd rectal cases (n = 13), this observation should be regarded as hypothesis-generating; nonetheless, it is consistent with growing evidence linking adiposity to colorectal carcinogenesis and to specific molecular subtypes [53,54]. Whether obesity contributes to the development of MMRd rectal cancer or merely reflects the younger age structure of this subgroup cannot be determined from our data and warrants confirmation in larger series.

The clinical significance of this characterization is amplified by a rapidly evolving therapeutic landscape. Neoadjuvant anti–PD-1 immunotherapy has demonstrated extraordinary efficacy in MMRd/MSI-H rectal cancer, with pathological complete response rates markedly exceeding those achievable with standard chemoradiation, and has enabled organ-preserving strategies, including non-operative management, with durable oncological outcomes in select patients [55,56]. These developments have transformed MMR status from a prognostic biomarker into a treatment-defining criterion in locally advanced rectal cancer, making accurate and timely MMR classification at diagnosis both a clinical imperative and a prerequisite for multidisciplinary treatment planning. The rarity of MMRd in rectal tumors (4.1%) means that prospective evidence specific to this subgroup remains limited, and current management algorithms are largely extrapolated from metastatic MMRd or MMRd colonic data. The clinicopathological profile we have defined, younger patients and enrichment for LS-associated disease, provides a practically useful framework for preoperative identification of candidates for immunotherapy-based organ preservation and supports the systematic integration of MMR testing into the standard diagnostic workup of all rectal cancer patients, irrespective of clinical stage or family history. Beyond diagnostic performance, the translation of universal MMR screening into routine practice depends on practical implementation factors. IHC-based screening is less resource-intensive than downstream molecular testing, rapid (typically <48 h), and already integrated into standard pathology workflows, making first-line MMR testing broadly feasible even in resource-limited settings [57,58]. The subsequent steps of the algorithm—BRAF/MLH1 methylation testing and germline multigene panel sequencing—require greater laboratory infrastructure and longer turnaround times, and their availability, rather than IHC itself, is likely the principal barrier to universal implementation outside high-resource tertiary centers. A formal cost-effectiveness analysis was beyond the scope of this study but represents an important direction for future research, particularly in healthcare systems with more constrained genetic testing capacity.

### Limitations

Several limitations of this study warrant acknowledgement. First, the retrospective tertiary single-center design may limit generalizability to other healthcare settings, although the large consecutive cohort and prospective institutional database maintenance substantially mitigate ascertainment and selection bias. As a tertiary referral center with an active hereditary CRC surveillance program, our institution may preferentially attract patients with a stronger hereditary predisposition or more complex clinical presentations, potentially inflating the observed prevalence of MMRd and LS relative to non-referral or community settings; conversely, standardized surgical selection criteria at a high-volume center may reduce heterogeneity in tumour stage distribution compared with less selected populations. Second, MMR testing was unavailable in 14.4% of patients overall, predominantly concentrated in the early study period; sensitivity analyses restricted to the 2018–2023 period, characterized by 94.8% UTS coverage, confirmed the robustness of primary findings, supporting their validity in the contemporary UTS era. Third, MLH1 promoter methylation analysis, while implemented as part of the institutional UTS workflow since 2015, was not uniformly available across the entire study period, which may have marginally affected MMR classification accuracy in earlier years. Fourth, germline testing was completed in 93.9% of newly referred patients (62 of 66); the small proportion of untested cases may result in a marginal underestimation of the true LS detection rate. In addition, 7 of 62 tested individuals (11.3%) carried a variant of uncertain significance; subsequent reclassification of these variants, as periodically updated by ClinGen InSiGHT, could marginally alter the reported LS detection rate over time. Fifth, the limited number of confirmed LS cases (n = 22) and MMRd rectal cancer cases (n = 13) constrain statistical power for subgroup comparisons and warrants cautious interpretation of multivariable associations within these subgroups. Sixth, the absence of survival data precludes assessment of the prognostic impact of MMR status across the clinicopathological subgroups characterized in this study and represents a priority for future analyses. Seventh, this study did not capture downstream clinical outcomes following LS identification, including surveillance uptake, cascade genetic testing among at-risk relatives, or resulting changes in oncological management; prospective tracking of these outcomes represents an important next step to demonstrate the full clinical impact of UTS-based LS detection. Eighth, the absence of external validation and the single-center Italian population, which is largely ethnically homogeneous, may limit extrapolation of these findings to other healthcare systems and more ethnically diverse populations, where MMRd/LS prevalence and referral patterns may differ. Finally, the comparison of hereditary syndrome burden between eoCRC and loCRC (12.8% vs. 1.8%) should be interpreted with caution, as germline testing was not applied uniformly across age groups: eoCRC patients were systematically referred for genetic counselling irrespective of MMR status, whereas older patients were typically tested only in the presence of MMRd or specific clinical suspicion. This differential ascertainment likely inflates the observed disparity and precludes an unbiased estimate of the true prevalence difference between age groups.

## 5. Conclusions

This large real-world cohort study provides several clinically actionable findings. UTS identifies a substantial proportion of Lynch syndrome carriers, including the majority of newly diagnosed cases, who would be missed by family history- or age-based referral criteria, confirming its role as the standard of care for LS detection in routine CRC practice. The relationship between age and MMRd prevalence proved more complex than a simple age-based dichotomy, with a borderline-significant association emerging only after adjustment for tumour location—underscoring that age-based selective testing cannot reliably substitute for universal MMR screening across all age groups. The broader hereditary burden in eoCRC, encompassing syndromes beyond LS, supports the integration of comprehensive multigene panel testing in younger patients irrespective of MMR status. Finally, MMRd rectal cancer, though rare, displays a distinct and younger clinicopathological profile that is likely enriched for LS-associated disease and is of direct therapeutic relevance in the era of immunotherapy-based organ preservation. Together, these findings support the standardization and expansion of UTS programs across all surgically treated CRC subgroups, anatomical sites, and disease stages.

## Figures and Tables

**Table 1:** Baseline characteristics of the colorectal cancer cohort.

Variable	Number (n)	Percent (%)
Total CRC cases	1022	100
Sex		
Male	576	56.3
Female	446	43.7
Age		
≤50 years (eoCRC)	117	11.4
51–69 years	373	36.5
≥70 years	532	52.1
Tumor location		
Proximal Colon	399	39.0
Distal Colon	230	22.5
Rectum	393	38.5
Distribution of cases by year		
2015–2017	336	32.8
2018–2020	334	32.6
2021–2023	352	34.4
UTS performed	875	85.6
Missing	147	14.4
Distribution of missing cases by year		
2015–2017	111	33.0 *
2018–2020	27	8.1 *
2021–2023	9	2.6 *

* Percentages for UTS not performed by period refer to the number of operated CRC patients within each period. Abbreviations: CRC, colorectal cancer; eoCRC, early-onset colorectal cancer; UTS, universal tumor screening.

**Table 2:** Clinicopathological characteristics of colorectal cancer patients according to MMR status.

Variable	MMRp (n = 736)	MMRd (n = 139)	*p* Value §
**Sex**			
**Male**	422 (57.3)	57 (41.0)	0.0004
**Female**	314 (42.6)	82 (58.9)	
**Age [Median (IQR)] (y)**	70 (58–82)	76 (61–85)	
**≤50 years**	76 (10.3)	19 (13.7)	0.001
**51–69 years**	283 (38.4)	29 (20.9)	0.0004
**≥70 years**	377 (51.2)	91 (65.5)	
**BMI [Median (IQR)] (kg/m^2^)**	24.8 (22.1–27.9)	24.5 (21.7–28.8)	
**≥30**	89 (12.1)	24 (17.3)	0.78
**<30**	641 (87.1)	109 (78.4)	0.07
**Missing**	6 (0.8)	6 (4.3)	
**Tumor location**			
**Proximal Colon**	248 (33.7)	110 (79.1)	<0.0001
**Distal Colon**	187 (25.4)	16 (11.5)	
**Rectum**	301 (40.9)	13 (9.4)	
**Tumor Stage**			
**0**	25 (3.4)	1 (0.7)	<0.0001
**I**	145 (19.7)	20 (14.4)	
**II**	206 (28.0)	68 (48.9)	
**III**	234 (31.8)	41 (29.5)	
**IV**	126 (17.1)	9 (6.5)	
**Grading**			
**Low**	495 (67.3)	54 (38.8)	<0.0001
**High**	166 (22.6)	72 (51.8)	
**Not applicable**	75 (10.2)	13 (9.4)	
**Vascular invasion**			
**Present**	383 (52.0)	71 (51.1)	0.85
**Absent**	353 (48.0)	68 (48.9)	
**Perineural invasion**			
**Present**	438 (59.5)	71 (51.0)	0.07
**Absent**	298 (40.5)	68 (48.9)	
**Family history (FDR)**			
**FDR**	112 (15.2)	35 (25.2)	0.0035
**Missing**	62 (8.4)	12 (8.6)	
**Family history (FDR plus)**			
**FDR plus**	13 (1.8)	11 (7.9)	<0.0001
**Missing**	62 (8.4)	12 (8.6)	

Abbreviations: MMRp, mismatch repair proficient; MMRd, mismatch repair deficient; BMI, body mass index; FDR, at least one first-degree family member with CRC; FDR plus, two or more affected relatives including at least one first-degree relative. § *p* values were calculated after excluding missing values.

**Table 3:** Univariate and multivariable analysis of factors associated with MMRd colorectal cancer.

Variable	Univariate Analysis			Multivariable Analysis		
	Odds Ratio §	95% CI	*p* Value	Odds Ratio §	95% CI	*p* Value
**Sex (female vs. male)**	1.93	1.34–2.78	0.0004	1.82	0.81–4.32	0.12
**Age (≥70 years vs. <70)**	1.80	1.23–2.61	0.002	2.41	1.04–5.63	0.04
**Side (Colon proximal-distal vs. Rectum)**	6.48	3.63–11.59	<0.0001	5.52	2.10–16.64	0.001
**Tumor Stage (0–II vs. III–IV)**	1.70	1.17–2.48	0.005	2.52	1.30–4.90	0.006
**Grading (High vs. Low)**	3.97	2.66–5.89	<0.0001	2.32	0.91–5.55	0.06
**Family history** **(FDR, yes vs. no)**	1.91	1.22–3.00	0.0035	1.53	0.52–4.75	0.39
**Family history** **(FDR plus, yes vs. no)**	4.82	2.09–11.10	<0.0001	3.42	0.41–28.43	0.22

Abbreviations: MMRd, mismatch repair deficient; CI, confidence interval; FDR, at least one first-degree family member with CRC; FDR plus, two or more affected relatives including at least one first-degree relative. § Odds ratios refer to the odds of MMRd status. Missing values were excluded from the corresponding analyses. The multivariable model included 721 patients with complete data on all covariates, including 115 MMRd events, corresponding to an events-per-variable ratio of 16.4 across the seven predictor terms.

**Table 4:** Clinicopathological features of sporadic MMRd and Lynch syndrome colorectal cancers.

Variable	Sporadic MMRd (n = 117)	Lynch Syndrome (n = 22)	*p* Value §
**Sex** **Male** **Female**	46 (39.3) 71 (60.7)	11 (50.0) 11 (50.0)	0.35
**Age [Median (IQR)] (y)** **≤50 years** **51–69 years** **≥70 years**	77 (70–83) 8 (6.8) 23 (19.7) 86 (73.5)	50.5 (41–63) 11 (50.0) 6 (27.3) 5 (22.7)	<0.0001 <0.0001
**BMI [Median (IQR)] (kg/m^2^)** **≥30** **<30** **Missing**	24.9 (22.0–28.4) 22 (18.8) 89 (76.1) 6 (5.1)	23.7 (21.2–26.3) 2 (9.1) 20 (90.9) 0 (0.0)	0.46 0.36
**Tumor location** **Proximal Colon** **Distal Colon** **Rectum**	98 (83.8) 8 (6.8) 11 (9.4)	12 (54.5) 8 (36.4) 2 (9.1)	0.001
**Tumor Stage** **0** **I** **II** **III** **IV**	0 (0.0) 16 (13.7) 58 (49.6) 36 (30.8) 7 (6.0)	1 (4.5) 4 (18.2) 10 (45.5) 5 (22.7) 2 (9.1)	0.25
**Grading** **Low** **High** **Not applicable**	46 (39.3) 61 (52.1) 10 (8.5)	8 (36.4) 11 (50.0) 3 (13.6)	0.65
**Vascular invasion** **Present** **Absent**	62 (53.0) 55 (47.0)	9 (40.9) 13 (59.1)	0.35
**Neural invasion** **Present** **Absent**	61 (52.1) 56 (47.9)	10 (45.5) 12 (54.5)	0.64
**Family history (FDR)** **FDR** **Missing**	27 (23.1) 10 (8.5)	8 (36.4) 2 (9.0)	0.17
**Family history (FDR plus)** **FDR plus** **Missing**	8 (6.8) 10 (8.5)	3 (13.6) 2 (9.0)	0.19

Abbreviations: MMRd, mismatch repair deficient; BMI, body mass index; FDR, at least one first-degree family member with CRC; FDR plus, two or more affected relatives including at least one first-degree relative. § *p* values were calculated after excluding missing values.

**Table 5:** Multivariable analysis of clinicopathological characteristics of sporadic and Lynch syndrome-associated MMRd tumours.

	Univariate Analysis			Multivariable Analysis		
	Odds Ratio	95% CI	*p* Value	Odds Ratio	95% CI	*p* Value
**Age (<70 years vs. ≥70 years)**	9.43	3.36–24.46	<0.0001	7.01	2.02–29.53	0.003
**Side (Distal colon/rectum vs. Proximal colon)**	4.29	1.66–11.71	0.004	1.54	0.34–6.17	0.58

Abbreviations: CI, confidence interval. The multivariable model included all 139 patients with MMRd CRC, including 22 LS-associated events and two predictor terms, corresponding to an events-per-variable ratio of 11.0.

**Table 6:** Comparison of clinicopathological features between early-onset (EoCRC) and late-onset colorectal cancer (LoCRC).

	EoCRC n = 117 (11.4%)	LoCRC n = 905 (88.6%)	*p* Value §
**Sex** **Male** **Female**	59 (50.4) 58 (49.6)	517 (57.1) 388 (42.9)	0.11
**Localization** **Proximal colon** **Distal colon** **Rectum**	30 (25.6) 33 (28.2) 54 (46.2)	369 (40.8) 197 (21.8) 339 (37.5)	0.007
**Stage** **0** **I** **II** **III** **IV**	3 (2.5) 26 (22.2) 31 (26.5) 37 (31.6) 20 (17.1)	43 (4.7) 181 (20.0) 277 (30.6) 272 (30.1) 132 (14.6)	0.59
**ASA score** **0–I** **II** **III–IV** **Missing**	25 (21.4) 70 (59.8) 11 (9.4) 11 (9.4)	26 (2.9) 433 (47.8) 391 (43.2) 55 (6.1)	<0.0001
**BMI [Median (IQR)] (kg/m^2^)** **≥30** **<30** **Missing**	24.0 (21.3–27.2) 13 (11.1) 103 (88.0) 1 (0.9)	24.9 (22.1–28.2) 129 (14.3) 747 (82.5) 29 (3.2)	0.18 0.31
**Grading** **Low** **High** **Not applicable**	79 (67.5) 23 (19.7) 15 (12.8)	571 (63.1) 247 (27.3) 87 (9.6)	0.15
**Vascular invasion** **Present** **Absent**	58 (49.6) 59 (50.4)	463 (51.2) 442 (48.8)	0.76
**Neural invasion** **Present** **Absent**	56 (47.9) 61 (52.1)	494 (54.6) 411 (45.4)	0.20
**Family history (FDR)** **FDR** **Missing**	22 (18.8) 14 (11.9)	130 (14.4) 77 (8.5)	0.14
**Family history (FDR plus)** **FDR plus** **Missing**	3 (2.6) 14 (11.9)	27 (2.9) 77 (8.5)	0.71
**MMRp** **MMRd** **MSI/MMR status Missing**	76 (65.0) 19 (16.2) 22 (18.8)	660 (72.9) 120 (13.3) 125 (13.8)	0.23
**Braf status among loss of MLH1 (only/and PMS2) (tot 116)** **Braf wt** **Braf mut** **Braf missing**	n = 6 5 (83.3) 1 (16.7) 0 (0)	n = 110 47 (42.7) 62 (56.4) 1 (0.9)	0.09

Abbreviations: EoCRC, early-onset colorectal cancer; LoCRC, late-onset colorectal cancer; ASA, American Society of Anesthesiologists; BMI, body mass index; MMRp, mismatch repair- proficient; MMRd, mismatch repair–deficient; MSI, microsatellite instability; wt, wild-type; mut, mutated; FDR, at least one first-degree family member with CRC; FDR plus, two or more affected relatives including at least one first-degree relative. § *p* values were calculated after excluding missing values.

**Table 7:** Comparison of clinicopathological features between MMRd colon and rectal cancers.

	MMRd Colon Cancer	MMRd Rectal Cancer	*p* Value §
MMRd	n = 126/561, 22.5%	n = 13/314, 4.1%	<0.0001
**Sex** **Male** **Female**	52 (41.3) 74 (58.7)	5 (38.5) 8 (61.5)	>0.99
**Age [Median (IQR)] (y)** **≤50 years** **51–69 years** **≥70 years**	77 (66–83) 14 (11.1) 27 (21.4) 85 (67.5)	67 (50–74) 5 (38.5) 2 (15.4) 6 (46.2)	0.003 0.03
**BMI [Median (IQR)] (kg/m^2^)** **≥30** **<30** **Missing**	24.2 (21.7–27.7) 18 (14.3) 102 (81.0) 6 (4.8)	26.4 (23.8–31.8) 6 (46.2) 7 (53.8) 0 (0.0)	0.23 0.01
**Stage** **0** **I** **II** **III** **IV**	1 (0.8) 18 (14.3) 62 (49.2) 37 (29.4) 8 (6.3)	0 (0.0) 2 (15.4) 6 (46.2) 4 (30.8) 1 (7.7)	>0.99
**Grading** **Low** **High** **Not applicable**	47 (37.3) 68 (54.0) 11 (8.7)	7 (53.8) 4 (30.8) 2 (15.4)	0.24
**Vascular invasion** **Present** **Absent**	62 (49.2) 64 (50.8)	9 (69.2) 4 (30.8)	0.24
**Neural invasion** **Present** **Absent**	64 (50.8) 62 (49.2)	7 (53.8) 6 (46.2)	>0.99
**Family history (FDR)** **FDR** **Missing**	32 (25.4) 11 (8.7)	3 (23.1) 1 (7.7)	>0.99
**Family history (FDR plus)** **FDR plus** **Missing**	10 (7.9) 11 (8.7)	1 (7.6) 1 (7.6)	>0.99

Abbreviations: MMRd, mismatch repair deficient; BMI, body mass index; FDR, at least one first-degree family member with CRC; FDR plus, two or more affected relatives including at least one first-degree relative. § *p* values were calculated after excluding missing values.

## Data Availability

The data supporting the findings of this study are available from the corresponding author upon reasonable request.

## Supplementary Materials

The following supporting information can be downloaded at https://www.mdpi.com/article/10.3390/cancers18162549/s1: Figure S1: AIFEG reference algorithm for universal screening–based Lynch syndrome identification; Table S1: Cohort overview, 2018–2023; Table S2: Clinicopathological characteristics by MMR status (tested cohort); Table S3: Sporadic MMRd vs. Lynch syndrome; Table S4: MMRd colon vs. rectal cancer; Table S5: Early-onset vs. late-onset CRC (full 2018–2023 cohort).

## Abbreviations

The following abbreviations are used in this manuscript:

BRAF mut	BRAF V600E mutated
BRAF wt	BRAF wild-type
BMI	Body mass index
CI	Confidence interval
CRC	Colorectal cancer
eoCRC	Early-onset colorectal cancer
FDR	First-degree relative
GPV	Germline pathogenic variant
IHC	Immunohistochemistry
loCRC	Late-onset colorectal cancer
LS	Lynch syndrome
MMR	Mismatch repair
MMRd	Mismatch repair–deficient
MMRp	Mismatch repair–proficient
MSI	Microsatellite instability
NGS	Next-generation sequencing
OR	Odds ratio
UTS	Universal tumor screening
