# Supplementary material for: Universal Tumor Screening in Colorectal Cancer: Role of MMR Immunohistochemistry for Lynch Syndrome and Early-Onset CRC
Source: Cancers (Basel). 2026 Aug 8;18(16):2549. doi: 10.3390/cancers18162549 (PMC13510323; doi:10.3390/cancers18162549)
Supplement: Supplementary file 1 [file cancers-18-02549-s001.zip › cancers-4427061-supplementary.pdf]

Supplementary Materials

Figure S1. AIFEG reference algorithm for universal screening–based Lynch syndrome identification. National diagnostic flowchart approved by the Italian Association for the Study of Familial and Hereditary Gastrointestinal Tumors (AIFEG), on which the institutional screening pathway of this study (Figure 1) is based. *Reproduced from Tibiletti MG, et al Universal testing for MSI/MMR status in colorectal and endometrial cancers to identify Lynch syndrome cases: state of the art in Italy and consensus recommendations from the Italian Association for the Study of Familial Gastrointestinal Tumors (A.I.F.E.G.). Eur J Cancer Prev. 2022 Jan 1;31(1):44-49. doi: 10.1097/CEJ.0000000000000677. PMID: 33767077.*

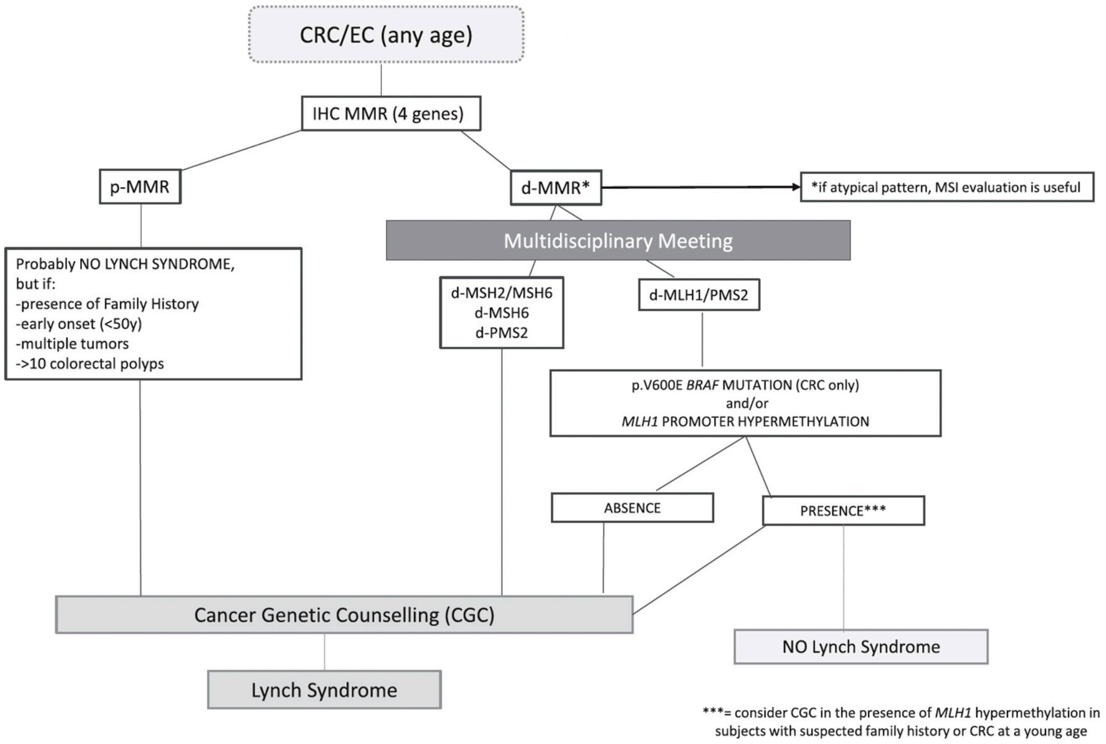

Flowchart approved by the A.I.F.E.G for the identification of Lynch syndrome cases through the universal screening. CGC, cancer genetic counseling; CRC, colorectal cancer; d-MMR, mismatch repair defective; EC, endometrial cancer; IHC, immunohistochemistry; MSI, microsatellite instability; p-MMR, mismatch repair proficient.

Sensitivity analysis restricted to patients operated between January 2018 and December 2023 (near-complete MMR testing coverage).

Table S1. Cohort overview, 2018–2023

Variable	Number (n)	Percent (%)
**Total CRC cases, 2018–2023**	**686**	**100**
**Distribution of cases by year**		
2018–2020	334	48.7
2021–2023	352	51.3
**UTS performed**	**650**	**94.8**
**MMR testing not performed**	**36**	**5.2**
**Distribution of missing cases by year**		
2018–2020	27	3.9
2021–2023	9	1.3

Percentages for missing cases by year refer to all operated CRC patients within each period.

Table S2. Clinicopathological characteristics by MMR status (tested cohort)

Variable	MMRp (n=542)	MMRd (n=108)	P value§
**Sex**			**0.038**
Male	305 (56.3)	49 (45.4)	
Female	237 (43.7)	59 (54.6)	
**Age [Median (IQR)] (y)**	**71 (59–81)**	**76 (67–84)**	**0.004**
≤50 years	62 (11.4)	8 (7.4)	0.0005
51–69 years	205 (37.8)	23 (21.3)	
≥70 years	275 (50.7)	77 (71.3)	
**BMI [Median (IQR)] (kg/m²)**	**24.9 (22.1–28.3)**	**25.8 (22.8–30.1)**	**0.21**
≥30	74 (13.7)	23 (21.3)	0.030
<30	463 (85.4)	81 (75.0)	
Missing	5 (0.9)	4 (3.7)	
**Tumor location**			**<0.0001**
Proximal colon	180 (33.2)	87 (80.6)	
Distal colon	142 (26.2)	12 (11.1)	
Rectum	220 (40.6)	9 (8.3)	
**Tumor stage**			**0.013**
0	15 (2.8)	2 (1.9)	
I	112 (20.7)	14 (13.0)	
II	165 (30.4)	50 (46.3)	
III	170 (31.4)	33 (30.6)	
IV	80 (14.8)	9 (8.3)	
**Grading**			**<0.0001**
Low	350 (64.6)	45 (41.7)	
High	130 (24.0)	51 (47.2)	
Not applicable	62 (11.4)	12 (11.1)	

**Vascular invasion**			**0.276**
Present	270 (49.8)	60 (55.6)	
Absent	272 (50.2)	48 (44.4)	
**Perineural invasion**			**0.504**
Present	310 (57.2)	58 (53.7)	
Absent	232 (42.8)	50 (46.3)	
**Family history (FDR)**			**0.015**
FDR+	92 (17.0)	29 (26.9)	
FDR–	405 (74.7)	70 (64.8)	
Missing	45 (8.3)	9 (8.3)	
**Family history (FDR plus)**			**0.003**
FDR plus +	14 (2.6)	9 (8.3)	
FDR plus –	483 (89.1)	90 (83.3)	
Missing	45 (8.3)	9 (8.3)	

*Data are n (%) unless otherwise indicated. § P values calculated after excluding missing/not-assessable values; categorical variables compared by χ^2 or Fisher's exact test as appropriate to expected cell counts; * Wilcoxon rank-sum test for continuous variables. Stage X (unknown) excluded from the stage P value.*

Table S3. Sporadic MMRd vs Lynch syndrome

**Variable**	**Sporadic MMRd (n=91)**	**Lynch Syndrome (n=17)**	**P value§**
**Sex**			**1.00**
Male	41 (45.1)	8 (47.1)	
Female	50 (54.9)	9 (52.9)	
**Age [Median (IQR)] (y)**	**78 (72–84)**	**52 (45–66)**	**<0.0001**
≤50 years	1 (1.1)	7 (41.2)	<0.001
51–69 years	17 (18.7)	6 (35.3)	
≥70 years	73 (80.2)	4 (23.5)	
**BMI [Median (IQR)] (kg/m²)**	**25.9 (23.0–30.2)**	**24.0 (21.8–27.4)**	**0.28**
≥30	21 (23.1)	2 (11.8)	0.35
<30	66 (72.5)	15 (88.2)	
Missing	4 (4.4)	0 (0.0)	
**Tumor location**			**0.41**
Proximal colon	75 (82.4)	12 (70.6)	
Distal colon	9 (9.9)	3 (17.6)	
Rectum	7 (7.7)	2 (11.8)	
**Tumor stage**			**0.41**
0	1 (1.1)	1 (5.9)	
I	11 (12.1)	3 (17.6)	
II	44 (48.4)	6 (35.3)	
III	28 (30.8)	5 (29.4)	
IV	7 (7.7)	2 (11.8)	
**Grading**			**0.78**
Low	39 (42.9)	6 (35.3)	
High	43 (47.3)	8 (47.1)	
Not applicable	9 (9.9)	3 (17.6)	
**Vascular invasion**			**0.29**
Present	53 (58.2)	7 (41.2)	
Absent	38 (41.8)	10 (58.8)	
**Perineural invasion**			**1.00**

Present	49 (53.8)	9 (52.9)	
Absent	42 (46.2)	8 (47.1)	
**Family history (FDR)**			**0.011**
FDR+	20 (22.0)	9 (52.9)	
FDR–	64 (70.3)	6 (35.3)	
Missing	7 (7.7)	2 (11.8)	
**Family history (FDR plus)**			**0.004**
FDR plus +	4 (4.4)	5 (29.4)	
FDR plus –	80 (87.9)	10 (58.8)	
Missing	7 (7.7)	2 (11.8)	

*Data are n (%) unless otherwise indicated. § P values calculated after excluding missing/not-assessable values; categorical variables compared by χ^2 or Fisher's exact test as appropriate to expected cell counts; * Wilcoxon rank-sum test for continuous variables. Stage X (unknown) excluded from the stage P value.*

Table S4. MMRd colon vs rectal cancer

Variable	MMRd Colon (n=99)	MMRd Rectal (n=9)	P value§
**MMRd prevalence**	**99/421 (23.5)**	**9/229 (3.9)**	**<0.0001**
**Sex**			**0.51**
Male	46 (46.5)	3 (33.3)	
Female	53 (53.5)	6 (66.7)	
**Age [Median (IQR)] (y)**	**78 (72–84)**	**64 (49–72)**	**0.006**
≤50 years	5 (5.1)	3 (33.3)	0.021
51–69 years	21 (21.2)	2 (22.2)	
≥70 years	73 (73.7)	4 (44.4)	
**BMI [Median (IQR)] (kg/m²)**	**25.4 (22.7–29.4)**	**28.2 (24.5–32.6)**	**0.18**
≥30	21 (21.2)	2 (22.2)	1.00
<30	74 (74.7)	7 (77.8)	
Missing	4 (4.0)	0 (0.0)	
**Tumor stage**			**0.20**
0	1 (1.0)	1 (11.1)	
I	12 (12.1)	2 (22.2)	
II	47 (47.5)	3 (33.3)	
III	30 (30.3)	3 (33.3)	
IV	9 (9.1)	0 (0.0)	
**Grading**			**1.00**
Low	42 (42.4)	3 (33.3)	
High	47 (47.5)	4 (44.4)	
Not applicable	10 (10.1)	2 (22.2)	
**Vascular invasion**			**0.73**
Present	54 (54.5)	6 (66.7)	
Absent	45 (45.5)	3 (33.3)	
**Perineural invasion**			**1.00**
Present	53 (53.5)	5 (55.6)	
Absent	46 (46.5)	4 (44.4)	
**Family history (FDR)**			**1.00**
FDR+	27 (27.3)	2 (22.2)	
FDR–	64 (64.6)	6 (66.7)	
Missing	8 (8.1)	1 (11.1)	
**Family history (FDR plus)**			**0.55**

FDR plus +	8 (8.1)	1 (11.1)	
FDR plus –	83 (83.8)	7 (77.8)	
Missing	8 (8.1)	1 (11.1)	

*Data are n (%) unless otherwise indicated. § P values calculated after excluding missing/not-assessable values; categorical variables compared by χ^2 or Fisher's exact test as appropriate to expected cell counts; * Wilcoxon rank-sum test for continuous variables. Stage X (unknown) excluded from the stage P value.*

Table S5. Early-onset vs late-onset CRC (full 2018–2023 cohort)

**Variable**	**EoCRC n=85 (12.4%)**	**LoCRC n=601 (87.6%)**	**P value§**
**Sex**			**0.039**
Male	39 (45.9)	347 (57.7)	
Female	46 (54.1)	254 (42.3)	
**Localization**			**0.001**
Proximal colon	18 (21.2)	252 (41.9)	
Distal colon	25 (29.4)	135 (22.5)	
Rectum	42 (49.4)	214 (35.6)	
**Stage**			**0.57**
0	1 (1.2)	3 (0.5)	
I	18 (21.2)	122 (20.3)	
II	22 (25.9)	190 (31.6)	
III	29 (34.1)	177 (29.5)	
IV	14 (16.5)	86 (14.3)	
X	1 (1.2)	23 (3.8)	
**ASA score**			**0.013**
0–I	20 (23.5)	100 (16.6)	
II	49 (57.6)	345 (57.4)	
III–IV	8 (9.4)	135 (22.5)	
Missing	8 (9.4)	21 (3.5)	
**Body mass index [median (IQR)]**	**24.1 (21.6–27.5)**	**25.0 (22.2–28.3)**	**0.22**
≥30	11 (12.9)	90 (15.0)	0.63
<30	73 (85.9)	490 (81.5)	
Missing	1 (1.2)	21 (3.5)	
**Grading**			**0.34**
Low	57 (67.1)	380 (63.2)	
High	18 (21.2)	160 (26.6)	
Not applicable	10 (11.8)	61 (10.1)	
**Vascular invasion**			**0.60**
Present	45 (52.9)	300 (49.9)	
Absent	40 (47.1)	301 (50.1)	
**Perineural invasion**			**0.056**
Present	38 (44.7)	335 (55.7)	
Absent	47 (55.3)	266 (44.3)	
**Family history (FDR)**			**0.067**
FDR+	18 (21.2)	83 (13.8)	
FDR–	58 (68.2)	467 (77.7)	
Missing	9 (10.6)	51 (8.5)	
**Family history (FDR plus)**			**1.00**
FDR plus +	7 (8.2)	53 (8.8)	
FDR plus –	69 (81.2)	497 (82.7)	
Missing	9 (10.6)	51 (8.5)	

**MMR/MSI status**			**0.31**
MMRp	62 (72.9)	480 (79.9)	
MMRd	8 (9.4)	100 (16.6)	
MSI/MMR status missing	15 (17.6)	21 (3.5)	
**BRAF status among loss of MLH1 only and/or PMS2**	**n=6**	**n=84**	**0.40**
BRAF wild-type	4 (66.7)	36 (42.9)	
BRAF mutated	2 (33.3)	47 (56.0)	
BRAF missing	0 (0.0)	1 (1.2)	

*Data are n (%) unless otherwise indicated. § P values calculated after excluding missing/not-assessable values; categorical variables compared by χ^2 or Fisher's exact test as appropriate to expected cell counts; * Wilcoxon rank-sum test for continuous variables. Stage X (unknown) excluded from the stage P value. The MMR/MSI status P value compares MMRd vs MMRp among tested patients (missing excluded).*
